# The Effect of Phytoestrogen on Thyroid in Subclinical Hypothyroidism: Randomized, Double Blind, Crossover Study

**DOI:** 10.3389/fendo.2018.00531

**Published:** 2018-09-11

**Authors:** Thozhukat Sathyapalan, Alison J. Dawson, Alan S. Rigby, Natalie J. Thatcher, Eric S. Kilpatrick, Stephen L. Atkin

**Affiliations:** ^1^Academic Diabetes, Endocrinology and Metabolism, Hull York Medical School, University of Hull, Hull, United Kingdom; ^2^Bradford Teaching Hospital NHS Foundation Trust, Bradford, United Kingdom; ^3^Hull York Medical School, University of Hull, Hull, United Kingdom; ^4^European Food Safety Authority, Parma, Italy; ^5^Department of Clinical Chemistry, Sidra Medical and Research Center, Doha, Qatar; ^6^Weill Cornell Medical College Qatar, Doha, Qatar

**Keywords:** subclinical hypothyroidisim, phytoestrogen, isoflavones, daidzein, genistein glucosides

## Abstract

**Objective:** Soy phytoestrogens are suggested to impair thyroid function but the effects of pharmacological doses of soy phytoestrogens are unknown; therefore, this study was performed to determine the effect of high dose soy phytoestrogens (66 mg) on thyroid function in subclinical hypothyroidism.

**Design and setting:** Randomized, double-blind, crossover study.

**Participants:** Forty four patients with subclinical hypothyroidism.

**Intervention:** Participants were randomly allocated to either 66 mg phytoestrogen with 30 g soy protein (active) or 0 mg phytoestrogen with 30 g soy protein (placebo) supplementation for 8 weeks, washed out for 8 weeks and then crossed over for another 8 week period.

**Main outcome measures:** The primary outcome was progression to overt hypothyroidism with the secondary outcome measures were changes in thyroid function tests.

**Results:** Two patients in this trial progressed into overt hypothyroidism after high dose phytoestrogen supplementation. TSH, free thyroxine and triiodothyronine did not differ between groups.

**Conclusion:** A pharmacological dose of 66 mg of soy phytoestrogens did not increase the overt thyroid failure rate or alter thyroid function tests in patients with subclinical hypothyroidism.

## Introduction

Soy foods is becoming increasingly popular in Western countries due to their potential health effects. Postulated health benefits of soy, due to their phytoestrogen components, include protection against ischemic heart disease (1–3), prostate and breast cancer (4–6), bone health (7), and alleviation of menopausal symptoms (8). Interest in the health benefits of phytoestrogens has led to the development of supplements containing phytoestrogen and the fortification of foods with soy phytoestrogens (9, 10). However, there are concerns that soy may adversely affect thyroid in susceptible individuals (11–13).

It is speculated that for certain subsets of the population soy isoflavones may be detrimental. Environmental factors are determinant for the appearance thyroid diseases in susceptible individuals. Dietary factors such as increased iodine intake, selenium, and vitamin D deficiency, exposure to radiation are environmental factors known to predispose to thyroid disorders (14). Goiter has been seen in infants fed soy formula; this is usually reversed by changing to cows' milk or iodine-supplemented diets (15, 16). *In vitro* research (17, 18) and in rodents (19, 20) supplemented with isoflavones suggests that soy can have a detrimental effect in thyroid. There are concerns that they may compromise thyroid function (18, 21), especially in patients with subclinical hypothyroidism.

In a double blind cross over study in patients with subclinical hypothyroidism with low dose phytoestrogen (30 g soy protein with 2 mg phytoestrogens, representative of Western diet), or a high dose phytoestrogen (30 g soy protein with 16 mg phytoestrogens, representative of a vegetarian diet), there was a threefold increased risk of developing overt hypothyroidism with high dose phytoestrogen supplementation (13).

Dietary intake of phytoestrogens in Asian diets has been estimated to be in the range of 30–50 mg/day of combined phytoestrogen aglycone equivalents (22, 23). Therefore, this double blind, cross over study was undertaken to assess the effect of a pharmacological dose of soy phytoestrogens with subclinical hypothyroidism who has compromised thyroid function. Soy protein was supplemented with 0 mg phytoestrogens or 66 mg phytoestrogens as a powder to be mixed with their daily food and is a similar dose used to treat menopausal symptoms.

## Patients and methods

Hull and East Yorkshire Ethics committee gave ethics approval for this study. Informed written consent was obtained from all study particpants before enrolling into the trial. Patients with subclinical hypothyroidism (TSH value between 4.8–10mU/L; reference range 0.5–4.7mU/L) with a free thyroxine (fT4) within the reference range were recruited. The screening thyroid function tests were done 4 to 8 weeks after the initial thyroid function test measurement. A total of 69 patients were initially identified. Patients taking drugs that might have interfered with thyroid function, anti-hypertensives, insulin sensitizing agents, lipid lowering medications, antibiotic use within the previous 6 months and women planning pregnancy were excluded. Patients who were taking vegetarian or vegan diets were excluded. Fifteen patients were excluded because they could not tolerate palatability of the soy preparation, thyroid function tests normalized for six patients suggesting transient thyroiditis, two patients had overt hypothyroidism, one patient was lost to follow up and another patient was already on thyroxine supplementation. Participants were instructed to maintain their current level of physical activity throughout the study. Participants were also instructed to avoid food products containing soy, mineral or vitamin supplementation and over the counter medications. Otherwise, they were encouraged to continue their usual diet. Plasma phytoestrogens were measured and dietary reinforcement was undertaken at each visit to ensure compliance.

## Study design

A double blind randomized, cross over trial was undertaken. A total of 44 participants (age range 23–80 years; 20 males, and 24 females) were randomized; 22 patients were commenced with the 0 mg phytoestrogen with soy protein powder (30 g) and 22 patients on the 66 mg phytoestrogen with soy protein powder (30 g) per day for 8 weeks to be mixed with their food (first phase). Participants received the alternative supplementation for 8 weeks (second phase) after an 8 week wash out period. Given that the half-life of thyroxine is around seven day, 8 week time period was chosen for each arm as this was the minimum duration of phytoestrogen exposure that might have been expected to affect thyroid function.

Participants received boxes of supplements containing the number of sachets required for 8 weeks of supplementation plus six reserve sachets in case of study material loss or any delay in their study visits.

After baseline tests, the participants were randomly allocated to either 66 mg phytoestrogen or 0 mg phytoestrogen supplementation, by a computer generated randomization list. Each randomization number corresponded with 1 of the 2 possible arms. Labeling of the identical study preparation was performed by personnel not involved in the study. Compliance was measured by counting the returned sachets.

**Figure 1:**
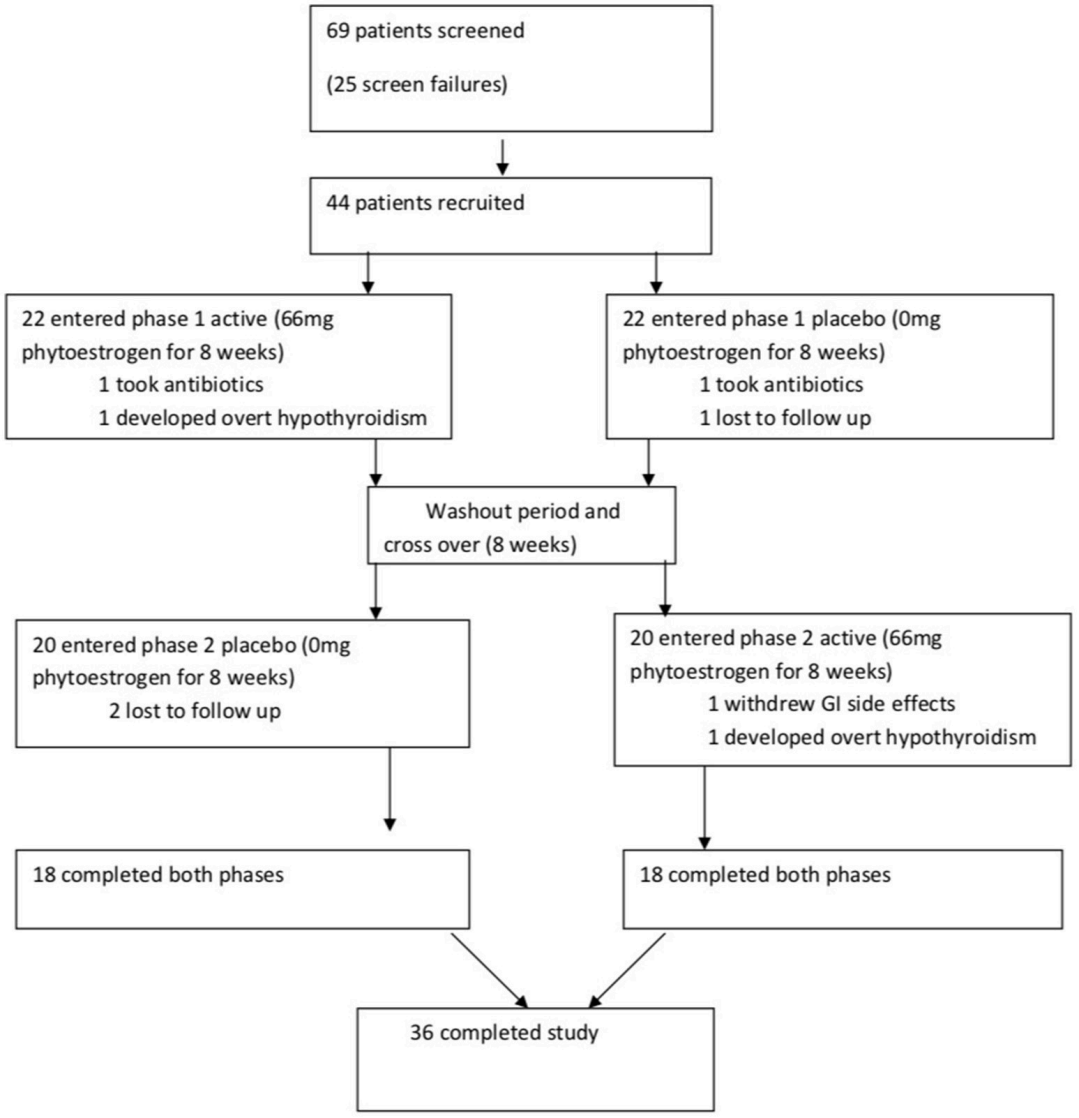
Flow chart of study.

The primary outcome of the trial was progression to overt hypothyroidism while secondary outcome measures were lipids, hsCRP, blood pressure and HOMA-IR. Overt hypothyroidism was defined as a combination of TSH > 4.7 mU/L and free thyroxine <9 pmol/L. The flow chart of participants is given in Figure 1.

## Phytoestrogen composition

The high-dose phytoestrogen preparation consisted of 30 g of soy protein (70% proteins) containing 66 mg of phytoestrogens and the isolated soy protein preparation contained 0 mg of phytoestrogens. Analysis of study material confirmed 12% glycitein, 35% daidzein, and 53% genistein. 10% of phytoestrogens were as aglycones or acetyl and malonyl glucosides forms whereas remaining 90% of phytoestrogens were in the primary glucoside form. Isolated soy protein preparation consisted of less than 300 parts per billion of isoflavones, achieved by serial alcohol washing (Dishman Ltd, India). This was confirmed analytically by FERA, Sand Hutton, York, UK. The phytoestrogens (Solgen40) and soy protein (SolconF) were supplied by Solbar Ltd, Israel, and was prepared by Essential Nutrition, Ltd, UK, who randomized the preparations.

## Study measurements

At the start and finish of each phase, following an overnight fast, blood pressure (BP) and weight were measured after which blood samples were collected. BP was measured after the participants had been seated quietly for at least 5 min with the arm supported at heart level. BP measurements were performed using (NPB-3900; Nellcor Puritan Bennett, Pleasanton, CA) during study visits. Fasting venous samples were collected, centrifuged at 2000 g at 4°C for 15 min. The aliquots stored at −80°C within 1 h of collection. Thyroid hormones were performed on an Abbott Architect i4000 immunoassay analyser (Abbott Diagnostics Division, UK). The reference range for TSH was (0.5–4.7 mU/L), free thyroxine (9–24 pmol/L) and fT3 was (2.5–5.3 pmol/L). Urinary iodine measurements in 24-h urine collections were undertaken by Inductively Coupled Plasma—Mass Spectrometry to monitor for iodine repletion. Phytoestrogen measurement was undertaken by Liquid Chromatography–Tandem Mass Spectrometry (HFL sport science laboratory, Cambridge, UK).

## Statistical analysis

For statistically significant reduction in fT4, a sample size of 40 participants in a cross over design gave 80% power to detect a mean decrease of 0.4nmol/L of free thyroxine, with a 2-sided alpha error of 0.05 (24).

Wilcoxon's signed-rank test was used to data that violated the assumptions of normality by the Kolmogorov-Smirnov test. The carry over and period effect that may have occurred were tested using the appropriate Student's *t*-test. Plasma phytoestrogen levels after wash out and after 0 mg/16 mg phytoestrogens were compared using independent samples *t*-test. Statistical analysis was performed using SPSS 14.0. The data is reported as mean ± SEM. We assumed an arbitrary level of 5% statistical significance (two-tailed).

## Results

Mean age of participant was 57.6 ± 1.8 years. 20 male and 24 female subjects with subclinical hypothyroidism participated in the study. Mean body mass index of patients was 30.4 ± 1.0 kg/m^2^, systolic blood pressure was 132.4 ± 3.2 mmHg and diastolic blood pressure of 80.9 ± 2.3 mmHg (Table 1). The marker for autoimmune thyroid dysfunction, TPO antibodies, was positive (>75u/mL) in 17 (38.6%) patients. Compliance was 98% in the study.

**Table 1:** Participant characteristics and effects on cardiovascular risk at the start of the trial and 8 weeks after supplementation.

**Soy protein and isoflavone (SPI)**				**Soy protein alone (SP)**			**SPI–SP**
	**Baseline**	**8 weeks**	***P*-value**	**Baseline**	**8 weeks**	***P*-value**	***p*-value**
Weight	87.6 ± 2.8	87.9 ± 2.9	0.39	87.0 ± 3.1	86.9 ± 3.0	0.56	0.28
BMI	30.1 ± 1.0	30.2 ± 1.0	0.34	30.4 ± 1.1	30.8 ± 1.1	0.57	0.30
SBP	134.0 ± 3.1	129.7 ± 2.8	0.10	132.0 ± 3.5	131.7 ± 3.2	0.91	0.74
DBP	83.5 ± 2.0	82.1 ± 2.4	0.59	80.6 ± 2.4	80.0 ± 2.0	0.75	0.12

*BMI, body mass index (kg/m^2)^; SBP, systolic blood pressure in (mmHg); DBP, diastolic blood pressure (mmHg)*.

Two participants (6%) developed overt hypothyroidism, both after high dose (66 mg) phytoestrogen. Both of them were TPO antibody negative. One of them was a 59 year old female with a baseline TSH of 6.8 mU/L and the other a 71 year old male subject with a baseline TSH of 7.1 mU/L. These two patients developed overt hypothyroidism during their study visit after 8 weeks phytoestrogen supplementation. They had a raised TSH of greater than 10 mIU/L, and a fT4 of less than 9pmol/L and were symptomatic. These two patients were started on levo-thyroxine, which was continued after 6 months since repeat thyroid function tests did not show over-replacement.

There was a significant change in daidzein and genistein (Table 2) after high-dose phytoestrogen and no changes after isolated soy protein supplementation. The mean TSH, fT4 and fT3 were not different after within or between groups comparing high dose or isolated soy protein supplementation (Table 2). There was no carry over or period effects in any of the parameters. There were no changes in TPO antibody positivity before or after either supplements.

**Table 2:** Thyroid function tests and serum isoflavone levels at the start and 8 weeks after supplementation.

**Soy protein and isoflavone (SPI)**				**Soy protein alone (SP)**			**SPI-SP**
	**Baseline**	**8 weeks**	***P* value**	**Baseline**	**8 weeks**	***P* value**	***P* value**
fT3	4.7 ± 0.2	4.7 ± 0.1	0.92	4.6 ± 0.2	4.3 ± 0.1	0.16	0.07
fT4	11.3 ± 0.3	11.9 ± 0.3	0.12	12.2 ± 0.3	12.0 ± 0.3	0.33	1.00
TSH	5.3 ± 0.6	5.5 ± 0.4	0.70	5.0 ± 0.3	5.2 ± 0.4	0.32	0.13
Daidzein	2.2 ± 0.4	41.0 ± 7.7	<0.01	2.4 ± 0.9	2.1 ± 0.6	0.40	<0.01
Genistein	2.6 ± 0.8	172.2 ± 40.4	<0.01	2.3 ± 0.6	2.4 ± 0.9	0.20	<0.01

*fT3, free triiodothyronine (T3) (pmol/L); fT4, free thyroxine (T4) (pmol/L); TSH, Thyroid Stimulating Hormone (mU/L); Daidzein (ng/ml); Genistein (ng/ml)*.

*There were no changes in anthropometric parameters including body mass index, waist circumference or hip circumference after 66 mg or 0 mg phytoestrogen supplementation*.

## Discussion

Following a pharmacological dose of soy phytoestrogens, there were no changes in thyroid function; however, two participants developed hypothyroidism after the high dose phytoestrogen phase, both of whom were TPO antibody negative. In a prospective study assessing the spontaneous course of hypothyroidism in females, 5.6% women per annum progressed from subclinical hypothyroidism to overt hypothyroidism (25) and therefore it was expected that in our study population one case per year would have progressed to overt hypothyroidism. Compared to the previous study using 2 mg and 16 mg phytoestrogen, it was expected that a larger number of patients (minimum of 3) would have developed overt hypothyroidism (26), suggesting that the pharmacological dose of soy phytoestrogens appeared not to confer a greater risk than the lower dose phytoestrogen levels.

Compared to the previous study using 2 mg and 16 mg phytoestrogen, it was expected that a larger number of patients would have developed overt hypothyroidism (13). This could be due to several reasons including relatively larger number of dropouts compromising the power of the study. Also there were more males (45.4%) in this study compared to 13% in the previous study (13). This is supported by community-based cross-sectional study in China which revealed intake of soy protein and isoflavones was inversely associated with number of cardiometabolic disturbances among women but not in men (27).

Another important factor which could have contributed to the results was that the patient group in this study was less sensitive to the hypothyroid effects of soy phytoestrogen as compared to the previous study (13) because of having low average TSH values (5.3 ± 0.6 mU/L in this study as compared to high serum TSH values (7.8 ± 0.4 mU/L) in the first part of study (13). As it has been shown previously that the level of serum TSH is an important risk factor for the development of overt hypothyroidism and increased risk in patients who have TSH values >6 mU/L as compared to patients who have TSH values < 6 mU/L (25, 28). 61% of participants had antibody negative subclinical hypothyroidism. We did not perform ultrasound of thyroid in the participants.

Isoflavones have shown to inhibit thyroid peroxidase which is an enzyme involved in the synthesis of T_3_ and T_4_ (18). Fischer rat thyroid cells (FRTL) when treated with soy protein and isoflavones; there was a dose dependent suppression of iodide uptake in FRTL cells whereas isoflavone alone was not effective. Soy protein and isoflavone increased non-glycosylated sodium/iodide symporter (NIS) and the 40 kDa thyroglobulin fragment which is a known autoimmunogen in the FRTL cells that potentially contributes to thyroid dysfunction (29).

We have previously reported that using this soy preparation with 66 mg of isoflavones caused a small though significant increase in TSH and decrease in fT4 in men and women with thyroid function tests within the reference range, though likely this would not be clinically significant (30, 31). The results of this study suggest that no further incremental deterioration in either TSH or fT4 occurs in compensated hypothyroidism.

In conclusion, it is reassuring that a pharmacological dose of 66 mg soy phytoestrogens was not associated with either a deterioration of thyroid function or an increased rate of thyroid failure over an 8 week period, above that associated with commonly ingested soy quantities.

## Ethics statement

Ethics approval was obtained from Hull and East Yorkshire Ethics committee. Informed written consent was obtained from each participant before enrolment in the study.

## Author contributions

TS, EK, NT, and SA conceived the study, TS and AD involved in conducting the study, AD and AR involved in statistical analysis, all authors involved in drafting and giving final approval of the manuscript.

### Conflict of interest statement

The authors declare that the research was conducted in the absence of any commercial or financial relationships that could be construed as a potential conflict of interest. The reviewer IR and handling Editor declared their shared affiliation.
